# Web-Based Exercise and Nutrition Intervention to Improve Leg Muscle Strength and Physical Functioning in Older Adults: Pre-Post Pilot Study

**DOI:** 10.2196/54392

**Published:** 2025-01-30

**Authors:** Berber Gijsbertha Dorhout, Nick Wezenbeek, Lisette C P G M de Groot, Pol Grootswagers

**Affiliations:** 1 Division of Human Nutrition and Health, Wageningen University & Research, Stippeneng 4, PO Box 17, Wageningen, 6700 AA, Netherlands; ^2^Research Group Innovation of Human Movement Care, HU University of Applied Sciences Utrecht, Utrecht, Netherlands; 3 CleverMove, Wageningen, Netherlands

**Keywords:** web-based lifestyle intervention, resistance exercise, protein intake, muscle strength, muscle mass, older adults, web-based exercise, nutrition, exercise, resistance training, sarcopenia

## Abstract

**Background:**

The lifestyle intervention ProMuscle*,* which combines resistance exercise and an increased protein intake, was effective in improving muscle strength, muscle mass, and physical functioning in older adults. However, due to a growing shortage of health care professionals, the rapidly growing aging population cannot be personally guided in the future. Therefore, Uni2Move, a scalable web-based variant of ProMuscle*,* was designed to reach larger groups of older adults without putting additional burden on health care professionals.

**Objective:**

The current study investigated the effects of a web-based lifestyle intervention on muscle strength, protein intake, and physical functioning in healthy older adults. In addition, we conducted a qualitative study to gather key insights of the participants involved, as little is known about older adults’ perceptions of web-based lifestyle interventions.

**Methods:**

A pre-post pilot study was conducted in the Netherlands. In the 24-week intervention, 19 healthy adults aged >55 years were included. They performed resistance training at home twice a week for 24 weeks via web-based workout videos. Videos (45‐60 minutes) were recorded by the fitness trainer and mainly focused on training leg muscles. In addition, older adults were advised on increasing protein intake via two web-based consultations by a dietitian in the first 12 weeks and via an e-learning course in the second 12 weeks. Intervention adherence and acceptance was measured in week 25. The 1 repetition maximum knee extension strength, repeated chair rise test, and protein intake were measured at baseline, week 13, and week 25. Linear mixed models were used to test differences over time. Semistructured interviews were used to gather experiences of participants. Atlas.ti version 22 was used to analyze the interviews.

**Results:**

The mean age of participants (n=19) at baseline was 69 (SD 7) years. The 1 repetition maximum knee extension strength and repeated chair rise test improved significantly during the 24-week intervention with a mean difference of 7.0 kg (95% CI 4.8-9.3; *P*<.001) and −1.2 seconds (95% CI −1.7 to −0.6; *P*<.001), respectively. Total protein intake per day did not change, whereas protein intake during breakfast had increased significantly after 13 weeks with a mean difference of 6.9 g (95% CI 1.1-12.7; *P*=.01). Qualitative research revealed that advantages of the program included no need to travel and exercising in their own environment. Disadvantages were the lack of physical interaction and no corrections by the trainer.

**Conclusions:**

The results of the web-based exercise and nutrition intervention Uni2Move indicate potential improvements of muscle strength and physical functioning in healthy middle-aged and older adults. Providing such lifestyle interventions on the internet could reach an increased number of older adults, providing the opportunity to contribute to the health and independence of the rapidly growing aging population.

## Introduction

The aging population is growing and life expectancy is increasing [1]. With advancing age, many people are experiencing sarcopenia. Sarcopenia can be defined as the age-related decline in skeletal muscle mass and function, highlighting the importance of advancing age as a factor of sarcopenia [2]. Sarcopenia is a natural part of the aging process, but its progression can be hastened by several factors, such as inflammation associated with aging (inflammaging), chronic diseases, lack of physical activity, inadequate nutrition, unintentional weight loss, and disuse events [3]. As the global population of older adults increases, the prevalence of sarcopenia is anticipated to grow accordingly [2,4].

As a consequence, the demand for care and support is increasing, whereas at the same time, the shortage of health care personnel is growing [5]. Governments worldwide have called for novel public health approaches concerning healthy aging, in response to the demographic shift and limited availability of health care professionals in the future [3,6]. Therefore, it is important to focus on the prevention of sarcopenia, with the goal of improving the health of the aging population and adding life to years [7].

Fortunately, lifestyle and behavioral interventions may reduce the prevalence of sarcopenia. The recommended lifestyle advice for treatment of sarcopenia consists of an increased dietary protein intake [8,9] and progressive resistance exercise [10,11]. The ProMuscle intervention, which combines resistance exercise with an increased protein intake, has been shown to be effective in improving muscle health of older adults in various settings [12-14]. In view of the rapidly growing aging population and the potential of digitalization in today’s world, a web-based version of ProMuscle seems promising. For that purpose, ProMuscle was transformed to a web-based program: Uni2Move.

Several studies showed that web-based exercise training (ie, participants train at home) resulted in an improved physical performance, physical activity, balance, mobility, and muscle strength in older adults [15,16]. In addition, a recent study showed that a blended home-based exercise and dietary protein intervention resulted in improved gait speed, physical activity level, muscle mass, strength, and dietary protein intake in older adults [17]. However, it is unknown whether an intervention combining resistance exercise and an increased protein intake that is offered completely on the internet is effective in improving muscle health. In addition, little is known about the perceptions and preferences of middle-aged and older adults regarding web-based interventions. Therefore, we investigated the effects of the web-based lifestyle intervention Uni2Move on muscle strength, physical functioning, and protein intake in healthy middle-aged and older adults and conducted qualitative research to gather the key insights and experiences from participants involved.

## Methods

### Uni2Move Program

The Uni2Move program was a 24-week web-based lifestyle intervention, based on the ProMuscle program.

#### Web-based resistance exercise training

Participants followed full body progressive resistance exercise training at home twice a week via web-based videos. Workouts lasted 45 to 60 minutes. The main focus was training the leg muscles, as these muscles are important in performing daily activities. At least 50% of each training session consisted of leg exercises, while the main upper body muscle groups were alternately trained once per week (shoulders, chest, back, and abdominal muscles).

The resistance training sessions comprised of a warm-up (5‐7 minutes), strength training (40‐50 minutes) and a cooldown (3‐5 minutes). The warm-up consisted of aerobic exercises, mobility exercises, and light strength exercises like air squats and push-ups against the wall. During the cooldown, light aerobic and dynamic stretching exercises were performed. The strength training session consisted of 4‐5 blocks. Each block consisted of 2‐4 unique exercises that were performed for 3‐4 sets of 40‐45 seconds. Each set of exercises was followed by a 20 second break. In total, 10‐12 unique exercises were performed per training session. An example of a block is as follows: set 1 squats, 20 second break, set 2 push-ups, 20 second break, set 3 squats, 20 second break, set 4 push-ups, etc. Effective muscle resting time was 80 seconds. This set-up was chosen as we expected long resting breaks to be detrimental for motivation and adherence in a home-based situation with only digital interaction possibilities.

Participants used their body weight, resistance bands, and weights (eg, dumbbells or bottles filled with sand). Exercises performed with bodyweight or weights were multiple variations of squats, box squats, lunges, wall-sits, bent-over rows, hip bridges, calf raises, planks, shoulder presses, lateral shoulder raises, reverse flies, push-ups, pullovers, crunches, and dips. Exercises performed with resistance bands were leg curls, leg extensions, leg abductions, glute kickbacks, knee lifts, horizontal rows, and shoulder external rotations. The exercise regimen was designed in a progressive manner by increasing the difficulty of the exercises during the 24-week period and encouraging participants to use higher weights, stronger bands, or to choose a more difficult variation of an exercise, which was provided by the trainer. In addition, more unilateral exercise variations were included from week 7 onward.

Although the main focus was on training around 12‐15 reps (2‐3 seconds for the eccentric movement and 1‐2 seconds for the concentric movement), a small number of exercises were performed isometrically (ie, wall-sit and plank) or with a focus on eccentric load (downward movement of a one-leg box squat). From week 13 onward, we introduced power training for the leg muscles in 50% of the trainings (ie, squats, lunges, and kettlebell swings) performed at high speed to stimulate improvements in physical functioning.

Training sessions were prerecorded by a fitness trainer and broadcasted at set times. A fitness trainer was present on the web at the start and end of the broadcast to welcome participants and answer questions. Participants could also perform a missed training at a time of their preference.

#### Increased protein intake

Before the start of the program, participants recorded their nutritional intake for 3 days in the Traqq app [18]. Based on their nutritional intake, participants received personal advice from the dietitian about increasing their protein intake up to 25 grams during breakfast, lunch, and dinner. A protein intake of 25 grams for each main meal was chosen because it has been shown to be the requirement for appropriate muscle protein synthesis [19]. A lower protein intake may attenuate the skeletal muscle protein synthesis response in older adults [20]. In addition, guidelines of the effective ProMuscle program, in which a dietary protein intake of 25 grams per main meal was advised, were used in the Uni2Move program [13]. Consultations took place via a web-based video call or by phone. During the evaluation consultation in week 6, the advice was adjusted if necessary. From week 13 onward, participants enrolled in a web-based nutrition module. The nutrition module consisted of 12 submodules, with a new submodule released every week. Various themes related to proteins were discussed in the form of videos, short texts, assignments, quizzes, and recipes. Each participant accessed the module with a personal account and performed it at their own pace.

The pilot study did not include a comparative control group of participants because of the previous ProMuscle studies performed. ProMuscle is an in-person program and has been proven to be effective in controlled and practice settings. This pilot study was an exploration to study the effects of ProMuscle when offered completely on the internet. In case of positive results, a larger study could be conducted including a larger study population and a control group.

### Participants

Adults aged 55 years and older living in and around Wageningen (The Netherlands) were recruited through flyers and news items in local newspapers. The participant’s eligibility for the web-based program was assessed through a physical screening; non-frail adults were allowed to participate. Participants’ ability to use the internet was assessed using two questions: (1) are you able to use email? (yes/no); (2) are you able to use internet explorer? (yes/no). Their ability to adjust the performance of exercises based on instructions was assessed by visual inspection of the fitness trainer to ensure safe participation. Participants were excluded during screening if participation might not be safe, in cases of medical limitations (such as severe heart failure or recovering from hip or knee arthroplasty), or in cases of an uncontrolled disease (ie, diabetes type 1 or 2, chronic obstructive pulmonary disease, hypertension, and cancer).

In total, 21 middle-aged and older adults were eligible for inclusion. After screening, 2 participants were excluded. Reasons for exclusion were (1) computer technical problems, resulting in them being unable to follow the web-based intervention and (2) not specified by the participant.

### Measurements and Analyses

#### Quantitative Measures

Questionnaires were used to collect baseline characteristics including age, sex, ethnicity, education level, care use, and sports. Body weight was measured to the nearest 0.1 kg using a digital scale and height was measured to the nearest 0.1 cm using a stadiometer. Measurements were performed at week 0, 13, and 25. Uni2Move was based on the effective ProMuscle program, which has a duration of 25 weeks, and effectiveness was measured at 0, 13, and 25 weeks [12]. In order to be able to compare Uni2Move (web-based program) to ProMuscle (in-person program) measurements of Uni2Move were also performed at the beginning, half way, and end of the program (0, 13, and 25 weeks). In addition, strength training needed to be performed for at least 6 months to assure maintenance of muscle strength and mass [21]. Muscle strength was measured by a fitness trainer with a 1-repetition maximum knee extension test using the leg extension machine. Physical performance was measured by the repeated chair rise test. The chair rise test was measured as the time to complete 5 chair rises with hands folded against the chest. The same chair was used during the measurements in week 0, 13, and 25. The measurements were performed according to a standardized protocol. In addition, fall frequency was measured using a questionnaire. Nutritional intake was measured for 3 days with the Traqq app in weeks 0, 13, and 25. The average protein intake over 3 days was calculated in gram per day, g/kg body weight/d and per meal moment (breakfast, lunch, or dinner).

#### Adherence

Training sessions were broadcasted using Webinargeek. The number of participants that watched the training session was tracked via Webinargeek. The percent adherence was calculated as the number of training sessions watched, divided by the number of training sessions that were provided, multiplied by 100. Adherence of the dietary consultations was registered by the dietitian for each participant separately. Engagement in the web-based nutrition module was registered for each submodule by the researchers by checking the percentage of participants that completed the submodule.

#### Acceptance

During the measurement in week 25, acceptance of the program was checked by asking the following question: “What grade do you give the Uni2Move program on a scale of 1‐10?” The question was asked for the total program and separately for the training sessions, as well as for the consultations with the dietitian and the nutrition module.

#### Qualitative Measures

A trained research assistant conducted semistructured interviews with participants to gain insight into experiences with the web-based program. Interview questions were based on a pretested interview guide from the ProMuscle in Practice Study and the guide was supplemented with questions specifically focusing on the web-based program [22]. The goal of the interview was to answer the following research questions:

1. How did participants experience the web-based training sessions?
2. How did participants experience the web-based dietary consultations?
3. How did participants experience the web-based nutrition module?

### Statistical Analyses

Univariate procedures were used to check for normal distribution of the data. Baseline data were expressed as means with SD or as percentages. Effects on muscle strength and physical performance were analyzed using linear mixed models in SPSS version 29 (IBM Corp). Fixed factors were age, sex, and measuring time. Statistical significance was indicated with a *P* value of <.05. Qualitative data were analyzed in Atlas.ti version 22 (Lumivero). Interviews were taped and transcribed verbatim, and transcripts from the interviews were coded using an inductive approach. First, the interviews were read to familiarize with the data. After that, parts of the transcript were identified and classified as open codes. Finally, repeating patterns were identified and sorted in themes.

### Ethical Considerations

The research team of the study officially consulted the medical ethical committee, with the question if medical ethical approval was necessary. No medical ethical approval was needed as this research did not fall within the remit of the Dutch Medical Research Involving Human Subjects Act (in Dutch: WMO; File number METC East-Netherlands: 2021‐13365). All steps in this study have been conducted ethically. Participants provided informed consent before participation [23]. Privacy was guaranteed by working via SmartPIA procedures. Participants were covered (for incidents related to the intervention) by the insurance of the organization. Study data were deidentified as every participant was assigned a participant number. Participants were not identifiable from participant numbers. Participants did not receive a compensation for the research. Since Uni2Move is a program that is supposed to be offered regularly, we asked participants to pay a set price of 157 euros (US $161.58) in total. We aimed to include older adults with a low socioeconomic position (SEP), by letting them participate for a reduced price of 30 euros (US $30.88) in total.

## Results

### Baseline Characteristics

A total of 21 community-dwelling older adults were eligible to participate, and 19 participants could be included. The mean age at baseline was 69 (SD 7) years and the majority were female (n=14, 74%; Table 1).

**Table 1.** Baseline characteristics of the intervention group (n=19).

Characteristic	Value
Age (years), mean (SD)	69 (7)
Sex, n (%)	
Female	14 (74)
Male	5 (26)
Height (cm), mean (SD)	168.4 (9.4)
Weight (kg), mean (SD)	71.4 (14.6)
Native Dutch ethnicity, n (%)	19 (100)
Education level, n (%)	
Low^a^	0 (0)
Intermediate^b^	2 (10)
High^c^	17 (90)
Receiving care^d^, n (%)	
Yes	1^e^ (5)
No	18 (95)
Doing sports weekly, n (%)	
Yes	15 (79)
No	4 (21)
Type of sports, n (%)	
Hiking	11 (58)
Cycling	9 (47)
Nordic walking	2 (10)
Running	1 (5)
Aqua gym	1 (5)
Swimming	1 (5)
Yoga	1 (5)
Tai chi	1 (5)
Dancing	1 (5)
Canoeing	1 (5)
Golf	1 (5)
Total exercise hours per week, mean (SD)	4 (2)

a Low: primary education, prevocational secondary education, secondary vocational education level 1, undergraduate senior general secondary education, or undergraduate preuniversity education.

b Intermediate: secondary vocational education level 2, 3, or 4; senior general secondary education; or preuniversity education.

c High: higher vocational education or university.

d Care was defined as informal care, housekeeping, personal care, nursing care, individual counseling, or group counseling.

e Care received was housekeeping, once a week.

### Quantitative Study

**Figure 1.**
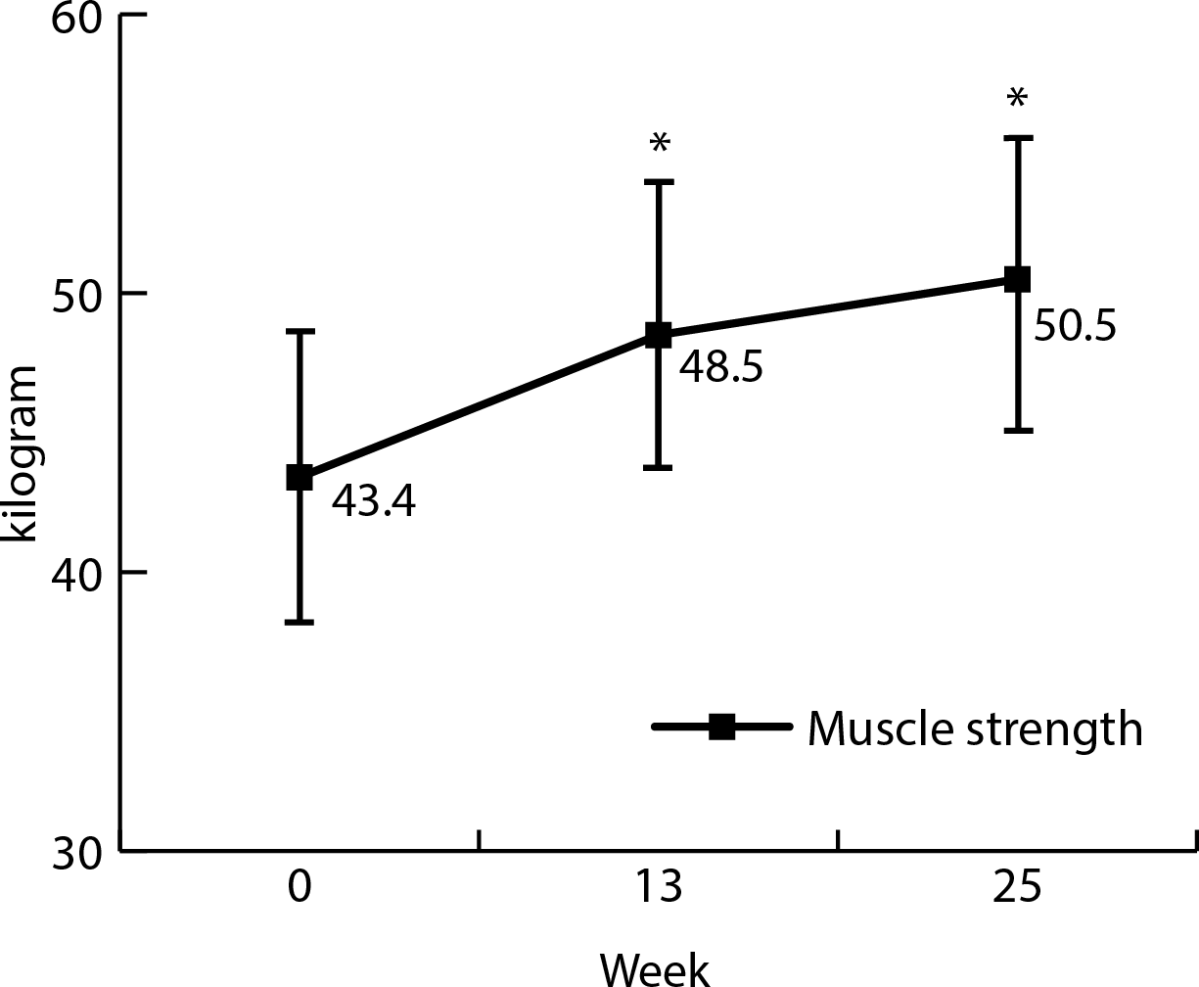
Effects of muscle strength in kilogram at week 0, 13, and 25. Error bars indicate SD. *Indicates a significant increase compared to baseline (*P*<.05).

Results are presented in Table 2 and Figures1  2. Participants significantly improved knee extension strength after 13 weeks by 5.1 kg (95% CI 2.8-7.3; *P*<.001) and by 7.0 kg (95% CI 4.8-9.3; *P*<.001) after 25 weeks, compared to baseline. Performance on the repeated chair test improved significantly after 13 weeks (−0.9 seconds, 95% CI −1.5 to −0.3; *P*=.001) and 25 weeks (−1.2 seconds, 95% CI −1.7 to −0.6; *P*<.001). Frequency of falls in the past 6 months decreased from 3 out of 17 (18%) at baseline to 1 out of 17 (6%) in weeks 13 and 25. Total protein intake did not change significantly between baseline and week 13 or week 25. Protein intake during breakfast increased significantly by 6.9 g (95% CI 1.1-12.7; *P*=.01) between week 0 and 13. Mean differences of protein intake during other meal moments did not change significantly.

**Figure 2.**
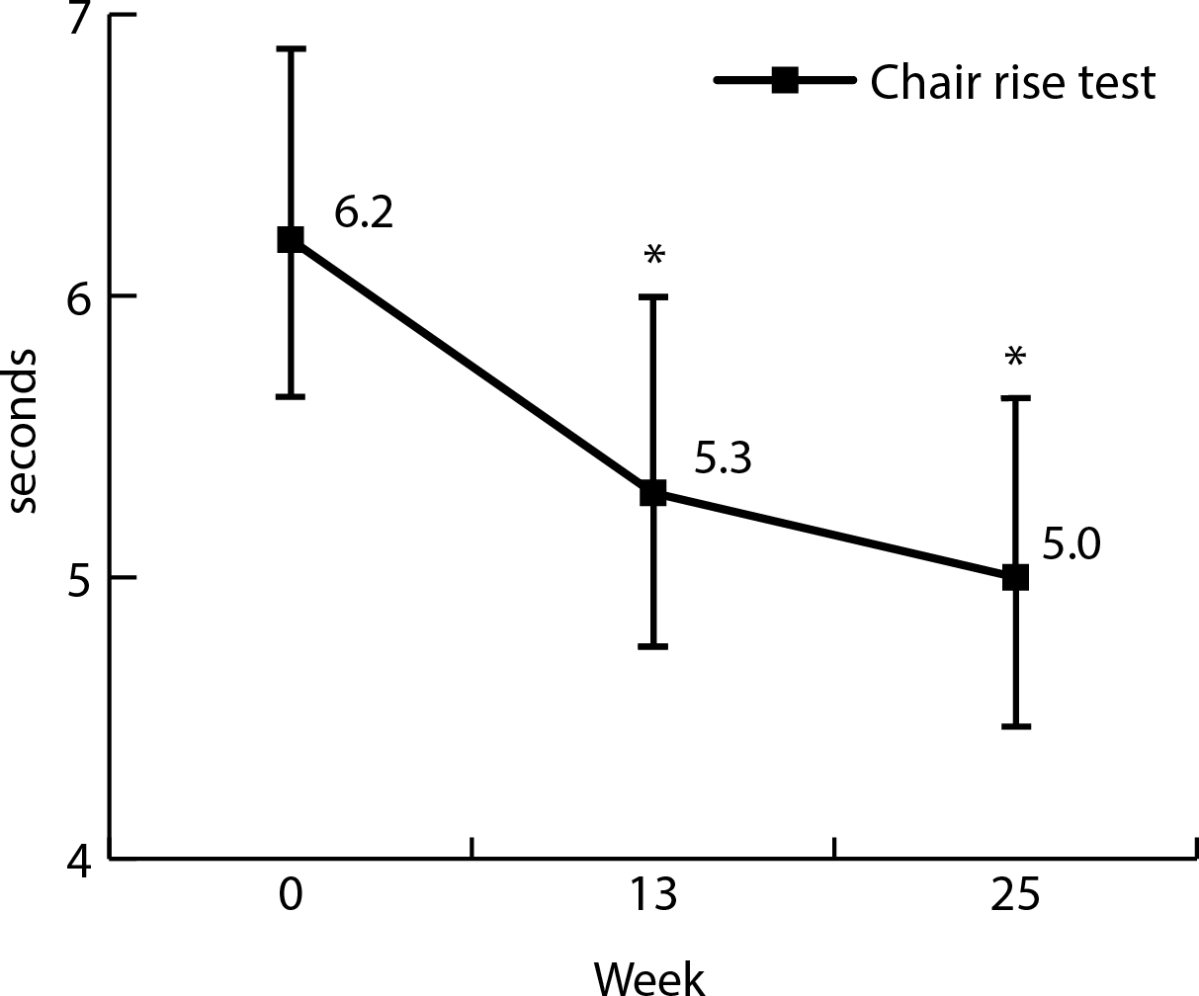
Effects of repeated chair rise test in seconds at week 0, 13, and 25. Error bars indicate SD. *Indicates a significant increase compared to baseline (*P*<.05).

**Table 2.** Study outcomes at week 0, 13, and 25 and the time effects of weeks 0‐25, 0‐13, and 13‐25.

Study outcome	Week 0, mean (95% CI)	Week 13, mean (95% CI)	Week 25, mean (95% CI)	Week 0‐25		Week 0‐13		Week 13‐25	
				Mean difference (95% CI)	*P* value	Mean difference (95% CI)	*P* value	Mean difference (95% CI)	*P* value
Chair rise test (s)	6.2 (5.6-6.9)	5.3 (4.7-6.0)	5.0 (4.4-5.7)	−1.2 (−1.7 to −0.6)	<.001	−0.9 (−1.5 to −0.3)	.001	−0.3 (−0.9 to 0.3)	.65
1RM^a^ knee extension (kg)	43.4 (38.5-48.3)	48.5 (43.6-53.4)	50.5 (45.5-55.4)	7.0 (4.8-9.3)	<.001	5.1 (2.8-7.3)	<.001	2.0 (−0.3 to 4.2)	.10
Protein intake (g/day)**	79.4 (65.6-93.2)	90.2 (75.9-104.4)	85.2 (70.3-100.2)	5.9 (−12.6 to 24.3)	>.99	10.8 (−6.9 to 28.5)	.39	−4.9 (−23.5 to 13.7)	>.99
Protein intake (g/kg body weight/day)	1.2 (0.9-1.5)	1.3 (1.0-1.6)	1.2 (0.9-1.5)	0.1 (−0.2 to 0.4)	>.99	0.2 (−0.1 to 0.4)	.54	−0.1 (−0.4 to 0.2)	>.99
Protein intake breakfast (g/day)	16.1 (10.9-21.3)	23.0 (17.6-28.4)	20.3 (14.6-26.1)	4.2 (−2.1 to 10.5)	.32	6.9 (1.1-2.7)	.01	−2.7 (−9.2 to 3.8)	.94
Protein intake lunch (g/day)	22.0 (17.1-27.0)	25.5 (20.0-31.0)	28.6 (22.9-34.4)	6.6 (−0.4 to 13.6)	.07	3.5 (−3.3 to 10.3)	.65	3.1 (−4.4 to 10.6)	.95
Protein intake dinner (g/day)	33.6 (25.9-41.4)	37.6 (29.2-45.9)	35.1 (26.3-44.0)	1.5 (−8.6 to 11.6)	>.99	3.9 (−5.6 to 13.5)	.96	−2.5 (−13.2 to 8.3)	>.99

a 1RM: 1 repetition maximum.

### Adherence

Participants followed on average 72% of the training sessions that were offered in the 24-week program. In the first 12 weeks, participants followed 77% of the training sessions, as compared to 67% in the second 12 weeks. The percentage of participants that participated in the web-based nutrition module, consisting of 12 modules, differed per week. On average, 80% of the participants performed the first 6 modules, and on average, 60% of the participants conducted the last 6 modules.

### Qualitative Study

Participants’ experiences were retrieved with interviews (n=7). The average length of the interviews was 27 minutes. For the interviewees, 6 out of 7 (86%) were woman with an average age of 66 years. The focus of the interviews was gaining insights in the experiences with the web-based training sessions, consultations, and nutrition module. Participants rated the total Uni2Move program 8.5 on average (scale 1‐10).

### Experiences With Web-Based Training Sessions

The training sessions were rated 8.6 on average (scale 1‐10). Most of the participants did not follow the web-based training sessions live but caught up at a time of their preference. They indicated several advantages of a web-based program, such as no need to travel, exercising in their own environment, and the possibility to train at a self-determined time. Participants appreciated that the fitness trainer provided variations to exercises, for example receiving an alternative exercise if they had a prosthesis.

The benefit is that you have no travel time of course. All I have to do here is walk from one room to another.

[Participant, female]

Disadvantages mentioned by participants were the lack of physical interaction, social control, training in a group, and corrections by the trainer. Some participants also indicated that more discipline was needed.

You need a little more discipline to think: tomorrow morning I am going to do it.

[Participant, female]

A drawback is that there is a little bit less correction. You need to think by yourself; How do I place my back and how do I stand? There is nobody who says; Hello, a bit to the front, a bit to the back.

[Participant, female]

### Experiences With Web-Based Dietary Consultations

Participants rated the consultations 7.3 on average (scale 1‐10). Participants appreciated the fact that the consultations with the dietitian could be planned at any time of day. In addition, the form of the consultation was flexible and participants mentioned that the dietitian always checked whether the information they provided was sufficient.

She always ended the consultation with asking whether the advise she provided was clear and sufficient, and for me it was fulfilling.

[Participant, female]

For several participants, the web-based consultations made the contact with the dietitian less personal, leaving limited room for discussion. In addition, the consultations were a challenge for a minority of the participants. Due to limited digital skills, video calling was challenging for them. As an alternative, these participants conducted phone consultations.

### Experiences With the Web-Based Nutrition Module

The web-based nutrition module was rated 7.1 on average (scale 1‐10). Participants mentioned that the level of difficulty for the modules was suitable. However, some participants thought it should be more challenging. Participants appreciated the schedule of the nutrition module, with a new module being presented on the web every week. In addition, the different elements of the nutrition module (knowledge clips, text, quizzes, and recipes) were mentioned as assets.

Participants missed the connection between the exercise and nutrition program, as they were offered separately and integration between the two was not present. According to the participants, a feature that should be added to the nutrition module is the possibility to have an web-based conversation in order to gather experiences from other users. On the content level, participants mentioned the need for information on skipping a meal, as well as vegetarian and vegan diets.

## Discussion

### Principal Findings

To our knowledge this is the first study evaluating the effects of an intervention combining resistance exercise and an increased protein intake that is completely web-based. Participants significantly improved knee extension strength after 13 weeks by 5.1 kg (95% CI 2.8-7.3; *P*<.001) and by 7.0 kg (95% CI 4.8-9.3; *P*<.001) after 25 weeks, compared to baseline. Performance on the repeated chair test improved significantly after 13 weeks by −0.9 seconds (95% CI −1.5 to −0.3; *P*=.001) and after 25 weeks by −1.2 seconds (95% CI −1.7 to −0.6; *P*<.001). Protein intake during breakfast increased significantly between week 0 and 13. Total protein intake per day did not change significantly. Participants positively valued Uni2Move, rating the total program an 8.5 (scale 1‐10). Advantages of the web-based training sessions and dietary consultations were the flexibility to conduct the program in their own environment and at a self-determined time. Challenges were the lack of personal contact and corrections by the trainer.

### Web-Based vs Offline Program

Results of the web-based Uni2Move program are in line with the offline program ProMuscle in Practice for the chair rise test after 13 weeks, which were −0.9 seconds (15%) versus −0.8 seconds (6%), respectively, and knee extension strength improved by 5.1 kg (12%) versus 9.6 kg (14%), respectively [13]. These findings show that the offline program is a promising strategy in improving muscle health of middle-aged and older adults. However, several challenges of the web-based program in comparison to the face-to-face program should be addressed. The average protein intake decreased after week 13, and the adherence dropped slightly during the course of the web-based program. Several participants indicated in the interviews that discipline was needed to proceed with the adjusted diet, and personal contact was lacking in the nutrition module. In line with this, Aalbers et al [10] concluded in their systematic review that tailored web-based interventions were rated as more interesting and helped middle-aged participants more, compared to generic web-based interventions [24]. This emphasizes the importance of personalizing web-based interventions and corresponding to the needs of participants. As suggested by participants, the web-based nutrition module can be improved by adding an option to chat with other participants. Social support is known to be an effective technique for the maintenance of a behavior change [25]. In addition, the nutrition module can be extended with advanced modules for extra challenge and additional information.

### Strengths and Limitations

First, a major strength of this study is the combination of quantitative data and qualitative data. In this way, insights on the effects of the program are combined with experiences of participants with the practicalities and feasibility of the program. Second, the limited costs of the intervention are a strength. Costs of preventive interventions are always challenging for professionals as well as participants. A web-based intervention limits costs by reducing travel motions, guiding large groups of participants during web-based training, reusing training videos, and reusing the nutrition module. This decreases a possible cost-related barrier for participation.

Several limitations should be mentioned. First, as participants perform the workout in their own environment, the performance of the exercises is less regulated. As a consequence, the intensity of the performed exercises could not be measured. On the other hand, the results show improvements for the chair rise test as well as the knee extension strength, and adherence levels and rating of the training sessions are satisfactory. Based on that, we may assume that participants performed the exercises with a sufficient intensity.

Second, the lack of physical attendance during the intervention limits the possibility of measuring adherence to the program in an accurate way. Attendance to training sessions was based on whether participants watched the video of the training session. In a face-to-face setting, the fitness trainer would be able to check who is present and actively participates in the training sessions. In addition, fitness trainers are not able to correct participants during at home training sessions. During the interviews, several participants also indicated the lack of guidance and corrections on the performance of exercises, whereas others did not miss the guidance. For older adults who live independently at home and without complex disorders, web-based guidance can be suitable. Physical guidance on site may be appropriate for frail older people who need more assistance. Further research is warranted to carefully assess eligibility of the program in different groups.

Third, the small study population is a limitation of this pilot study. In addition, this study population was highly educated for the most part. In general, adults with a higher education level have better health compared to their less-educated peers [26]. The latter group should also be included in future research to study the effects of Uni2Move on their muscle health and to contribute to reducing health inequalities. During the recruitment phase, several strategies that were shown to be successful strategies for reaching adults with a low SEP were executed in order to include participants with a lower SEP [24]. Multilayered recruitment strategies were conducted to increase engagement. First, we adjusted the recruitment material by adopting the language level and a personal approach by recruiting at low budget shops with flyers suitable for adults with low literacy levels. Second, we deployed existing networks and community networks by recruiting at the local food bank and neighborhoods where many adults with a low SEP live. Third, we tackled the common barrier of having limited resources to join the program by lowering the price of participation from 157 euros (US $161.58) to 30 euros (US $30.88) for participants with a low SEP. These strategies led to the inclusion of 2 participants with an intermediate education level. SEP was not measured because of participants’ privacy. Recently, Collombon et al [4] published a paper on recruitment strategies for reaching adults aged 50 years and older with low SEP for participation in web-based physical activity interventions [27]. Results show that recruitment via invitation letters administered by municipality was most promising; however, the costs were relatively high (>2000 euros; US $2058.34). This strategy could be deployed in future research to include a diverse group of participants.

### Future Directions

The main challenges of the web-based intervention include recruiting participants with a low SEP and adherence to the program. Recruitment of more participants with a low SEP should be attempted in follow-up studies by sending invitations via municipalities, as well as by focusing on recruitment on social media and at gyms [27]. Adherence to the program can be increased in several ways. Promising strategies appear to be increasing personal contact and tailoring the intervention to the needs of participants. This can be executed by conducting a hybrid intervention, alternating web-based and physical training sessions. In this way, fitness trainers can tailor the level of the exercises to the needs of the participants and can correct participants if needed. Personal contact evolves during training sessions, not only between participants but also between participants and fitness trainers. The advantages of a hybrid intervention are lower participation costs, flexibility of training sessions at home, less health care professionals needed, increased safety due to face-to-face training sessions, and expectedly higher adherence on the long term due to tailoring and personal contact.

### Conclusion

In summary, the web-based lifestyle intervention Uni2Move is a pilot study that resulted in an improvement of muscle strength and performance on the repeated chair rise test, comparable to the previous offline ProMuscle studies. The results should be interpreted with caution, as the study population is small. Advantages of the current intervention are flexibility for participants as well as professionals, as well as a lack of travel time and costs. Further research is needed to evaluate the intervention’s effects in larger groups, and should include participants with a low SEP, be in a hybrid form, and focus on adherence in the long term.

## Abbreviations

SEP: socioeconomic position

## References

[1] Shen Y, Liu D, Li S (2022). Effects of exercise on patients important outcomes in older people with sarcopenia: an umbrella review of meta-analyses of randomized controlled trials. Front Med (Lausanne).

[2] Tieland M, Dirks ML, van der Zwaluw N (2012). Protein supplementation increases muscle mass gain during prolonged resistance-type exercise training in frail elderly people: a randomized, double-blind, placebo-controlled trial. J Am Med Dir Assoc.

[3] (2024). Wet medisch-wetenschappelijk onderzoek met mensen. Overheid.nl.

[4] Collombon E, Bolman CAW, de Bruijn GJ, Peels DA, Lechner L (2024). Recruitment strategies for reaching adults aged 50 years and older with low socioeconomic status for participation in online physical activity interventions. Front Digit Health.

[5] van Dongen EJI, Haveman-Nies A, Doets EL, Dorhout BG, de Groot L (2020). Effectiveness of a diet and resistance exercise intervention on muscle health in older adults: ProMuscle in Practice. J Am Med Dir Assoc.

[6] (2024). Duurzame zorg en preventie (SPR 2019-2022). https://www.rivm.nl/over-het-rivm/strategisch-programma-rivm/duurzame-zorg-en-preventie.

[7] Kwasnicka D, Dombrowski SU, White M, Sniehotta F (2016). Theoretical explanations for maintenance of behaviour change: a systematic review of behaviour theories. Health Psychol Rev.

[8] Izquierdo M, Merchant RA, Morley JE (2021). International exercise recommendations in older adults (ICFSR): expert consensus guidelines. J Nutr Health Aging.

[9] van Dongen EJI, Doets EL, de Groot L, Dorhout BG, Haveman-Nies A (2020). Process evaluation of a combined lifestyle intervention for community-dwelling older adults: ProMuscle in Practice. Gerontologist.

[10] Aalbers T, Baars MAE, Rikkert M (2011). Characteristics of effective Internet-mediated interventions to change lifestyle in people aged 50 and older: a systematic review. Ageing Res Rev.

[11] (2024). Ouderen. Statistiek CB voor de.

[12] Coletta G, Phillips SM (2023). An elusive consensus definition of sarcopenia impedes research and clinical treatment: a narrative review. Ageing Res Rev.

[13] Paddon-Jones D, Rasmussen BB (2009). Dietary protein recommendations and the prevention of sarcopenia. Curr Opin Clin Nutr Metab Care.

[14] Lucassen DA, Brouwer-Brolsma EM, van de Wiel AM, Siebelink E, Feskens EJM (2021). Iterative development of an innovative smartphone-based dietary assessment tool: Traqq. J Vis Exp.

[15] Cruz-Jentoft AJ, Bahat G, Bauer J (2019). Sarcopenia: revised European consensus on definition and diagnosis. Age Ageing.

[16] (2020). UN decade of healthy ageing plan of action 2021-2030. https://cdn.who.int/media/docs/default-source/decade-of-healthy-ageing/decade-proposal-final-apr2020-en.pdf.

[17] Hill KD, Hunter SW, Batchelor FA, Cavalheri V, Burton E (2015). Individualized home-based exercise programs for older people to reduce falls and improve physical performance: a systematic review and meta-analysis. Maturitas.

[18] Katsanos CS, Kobayashi H, Sheffield-Moore M, Aarsland A, Wolfe RR (2005). Aging is associated with diminished accretion of muscle proteins after the ingestion of a small bolus of essential amino acids. Am J Clin Nutr.

[19] (2016). Global strategy on human resources for health: workforce. https://www.who.int/publications/i/item/9789241511131.

[20] Dorhout BG, de Groot L, van Dongen EJI, Doets EL, Haveman-Nies A (2022). Effects and contextual factors of a diet and resistance exercise intervention vary across settings: an overview of three successive ProMuscle interventions. BMC Geriatr.

[21] Bautmans I, Van Puyvelde K, Mets T (2009). Sarcopenia and functional decline: pathophysiology, prevention and therapy. Acta Clin Belg.

[22] Raghupathi V, Raghupathi W (2020). The influence of education on health: an empirical assessment of OECD countries for the period 1995-2015. Arch Public Health.

[23] Coelho-Júnior HJ, Milano-Teixeira L, Rodrigues B, Bacurau R, Marzetti E, Uchida M (2018). Relative protein intake and physical function in older adults: a systematic review and meta-analysis of observational studies. Nutrients.

[24] Nelson ME, Layne JE, Bernstein MJ (2004). The effects of multidimensional home-based exercise on functional performance in elderly people. J Gerontol A Biol Sci Med Sci.

[25] Rosenberg IH (1997). Sarcopenia: origins and clinical relevance. J Nutr.

[26] Johnson NR, Kotarsky CJ, Mahoney SJ (2022). Evenness of dietary protein intake is positively associated with lean mass and strength in healthy women. Nutr Metab Insights.

[27] van den Helder J, Mehra S, van Dronkelaar C (2020). Blended home-based exercise and dietary protein in community-dwelling older adults: a cluster randomized controlled trial. J Cachexia Sarcopenia Muscle.

